# A Dichotomous Role for FABP7 in Sleep and Alzheimer’s Disease Pathogenesis: A Hypothesis

**DOI:** 10.3389/fnins.2022.798994

**Published:** 2022-06-30

**Authors:** Hope Needham, Grace Torpey, Carlos C. Flores, Christopher J. Davis, William M. Vanderheyden, Jason R. Gerstner

**Affiliations:** ^1^Department of Biology, Gonzaga University, Spokane, WA, United States; ^2^Department of Translational Medicine and Physiology, Elson S. Floyd College of Medicine, Washington State University, Spokane, WA, United States; ^3^Sleep and Performance Research Center, Elson S. Floyd College of Medicine, Washington State University, Spokane, WA, United States; ^4^Steve Gleason Institute for Neuroscience, Elson S. Floyd College of Medicine, Washington State University, Spokane, WA, United States

**Keywords:** BLBP, astrocyte, neurodegeneration, circadian, omega-3 fatty acid

## Abstract

Fatty acid binding proteins (FABPs) are a family of intracellular lipid chaperone proteins known to play critical roles in the regulation of fatty acid uptake and transport as well as gene expression. Brain-type fatty acid binding protein (FABP7) is enriched in astrocytes and has been implicated in sleep/wake regulation and neurodegenerative diseases; however, the precise mechanisms underlying the role of FABP7 in these biological processes remain unclear. FABP7 binds to both arachidonic acid (AA) and docosahexaenoic acid (DHA), resulting in discrete physiological responses. Here, we propose a dichotomous role for FABP7 in which ligand type determines the subcellular translocation of fatty acids, either promoting wakefulness aligned with Alzheimer’s pathogenesis or promoting sleep with concomitant activation of anti-inflammatory pathways and neuroprotection. We hypothesize that FABP7-mediated translocation of AA to the endoplasmic reticulum of astrocytes increases astrogliosis, impedes glutamatergic uptake, and enhances wakefulness and inflammatory pathways via COX-2 dependent generation of pro-inflammatory prostaglandins. Conversely, we propose that FABP7-mediated translocation of DHA to the nucleus stabilizes astrocyte-neuron lactate shuttle dynamics, preserves glutamatergic uptake, and promotes sleep by activating anti-inflammatory pathways through the peroxisome proliferator-activated receptor-γ transcriptional cascade. Importantly, this model generates several testable hypotheses applicable to other neurodegenerative diseases, including amyotrophic lateral sclerosis and Parkinson’s disease.

## Fatty-Acid Binding Proteins

Fatty acids are critically important in the functioning of all living organisms as they are an important energy source and serve as key regulators of cell signaling processes. Polyunsaturated fatty acids (PUFAs) have traditionally been known as a major structural component of cell membranes; however, they also regulate signaling pathways related to gene expression, growth, survival, inflammation, and metabolism (Saltiel and Kahn, 2001; Hotamisligil, 2006, 2017). PUFAs are particularly abundant in the brain and are estimated to comprise 50% of the total mass of neuronal membranes (Zérouga et al., 1991; Bourre et al., 1992). They are also hydrophobic, and therefore must be escorted through the cytoplasm by a lipid chaperone. Fatty acid binding proteins (FABPs) are a family of small (14–15 kDa) intracellular lipid chaperone proteins that reversibly bind the hydrophobic long chain of PUFAs (Furuhashi and Hotamisligil, 2008; Storch and Corsico, 2008) and transport them to many different cellular locations, thereby enhancing their ability to affect a wide range of cellular processes. Of note, FABPs transport fatty acids to the endoplasmic reticulum for signaling, trafficking, and membrane synthesis as well as to the nucleus for lipid-mediated transcriptional regulation (Furuhashi and Hotamisligil, 2008).

Fatty acid binding proteins were first discovered in the cytosol of intestinal mucosa, liver, and myocardium tissues (Ockner et al., 1972), and were subsequently identified in other cell types and tissues. FABPs have phylogenetically conserved homologs in mice (*Mus musculus*), fruit flies (*Drosophila melanogaster*), nematodes (*Caenorhabditis elegans*), and humans suggesting evolutionarily conserved cellular functions (Smathers and Petersen, 2011). There are currently ten known members of the mammalian FABP family, each exhibiting a unique expression profile (D’Anneo et al., 2020). However, there is reported overlap in the expression patterns of the different FABPs, with each FABP being expressed in multiple cell and tissue types. For example, heart FABP (H-FABP/FABP3), epidermal FABP (E-FABP/FABP5), and brain FABP (B-FABP/FABP7) are all present within the adult mammalian brain. FABP3 is primarily expressed in neurons, FABP5 is expressed in neurons and glia, and FABP7 is most abundantly expressed in astrocytes and precursor cells (Owada, 2008; Storch and Thumser, 2010).

**FIGURE 1:**
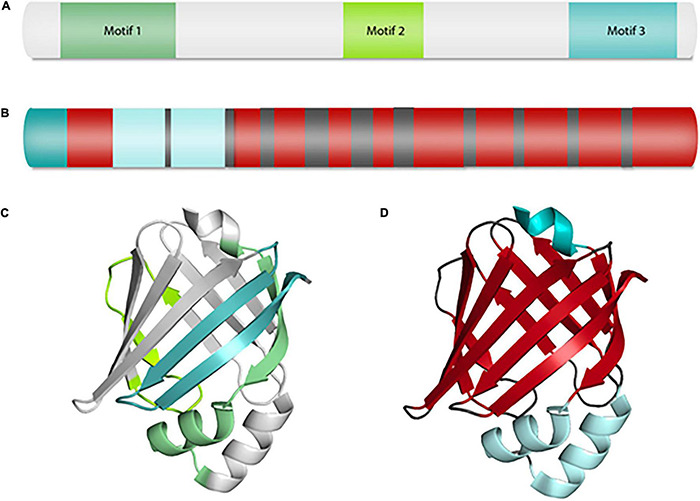
Fatty acid binding protein structure. FABP7 primary protein and ribbon structures displaying structural components common among all FABPs. **(A)** Representative primary protein structure showing the three distinct fatty acid binding motifs found in all FABPs. **(B)** Primary protein structure depicting the distribution of alpha helices (light blue and teal) and beta sheets (red). **(C)** Ribbon structure of FABP7 depicting the three conserved motifs shown in **(A)**. **(D)** Ribbon structure of FABP7 highlighting the placement of the ten-stranded beta barrel (red) and N-terminal helix-turn-helix motif forming a “cap” domain (light blue) shown in **(B)**. This figure depicts FABP7 (PDB code: 6L9O) and was created using PyMOL.

The sequence homology among FABPs ranges from 15 to 70%; however, the 3-dimensional structure is highly conserved (Chmurzyńska, 2006). All FABPs have three fatty acid binding motifs, a 10-stranded, antiparallel β-barrel structure (formed by two perpendicular, five-stranded β-sheets), a binding pocket inside of the β-barrel, and an N-terminal helix-turn-helix motif that forms the “cap” domain (Storch and Thumser, 2010; Figure 1). With the exception of liver FABP (FABP1), which can accommodate two fatty acids, all other FABPs bind one ligand at a time (Thompson et al., 1997). Despite variable sequence identities, FABPs display highly consistent binding patterns, with a direct correlation between FABP binding affinity and fatty acid hydrophobicity (Richieri et al., 2000). FABP structure-function studies have revealed that ligand binding triggers subtle conformational changes within FABPs that then strengthen different FABP-protein or FABP-membrane interactions (Storch and McDermott, 2009). For instance, FABP4, which binds multiple ligands with similar affinities, has been found to adopt different functional roles depending on the ligand bound and the conformational changes induced by each ligand (Gillilan et al., 2007).

## Fatty Acid Binding Protein 7

Fatty acid binding protein 7, also known as brain-type FABP, is ontologically expressed with a decrease in levels following normal lifespan (Clarke et al., 2018). FABP7 is abundant in radial glia during the mid-term embryonic stage of development and becomes primarily expressed in astrocytes and neural progenitors in adulthood (Owada et al., 1996; Owada, 2008). While the specific role of FABP7 remains unclear, it has been proposed to be important for cell growth and differentiation, with evidence suggesting that most neuronal cell populations are derived from FABP7-expressing progenitors (Feng et al., 1994; Anthony et al., 2004). Clinically, FABP7 has been implicated in a wide range of diseases including cancer, Down’s syndrome, schizophrenia, and the neurodegenerative diseases amyotrophic lateral sclerosis (ALS), Parkinson’s disease, and Alzheimer’s disease (AD; Sánchez-Font et al., 2003; Watanabe et al., 2007; Teunissen et al., 2011; De Rosa et al., 2012; Thumser et al., 2014; Islam et al., 2019). Here, we propose a FABP7-mediated cellular signaling model applicable to neurodegenerative disease that is supported by evidence derived from FABP7 studies in other diseases, particularly cancer.

## FABP7’s Dichotomous Role in Cancer

In the aggressive brain cancer glioblastoma, FABP7 expression is upregulated compared to healthy adult brain, and this increase has generally been associated with decreased survival times (Liang et al., 2005; Tso et al., 2006; De Rosa et al., 2012). Cancer progression has been proposed to be mediated not directly through FABP7 expression, but rather via the subcellular trafficking of fatty acids by FABP7 (Thumser et al., 2014). For example, in triple negative breast cancer, which also shows increased FABP7 expression, Alshareeda et al. (2012) found that FABP7 localization had prognostic implications, with nuclear FABP7-expressing tumors having significantly better prognosis than those with only cytoplasmic expression.

The differential functionality of FABP7 has been shown to be dependent upon the ratio of n-3:n-6 fatty acids (Mita et al., 2010). Although FABP7 can bind a variety of ligands, it has a strong affinity for n-3 PUFAs. Docosahexaenoic acid (DHA), an n-3 PUFA, and arachidonic acid (AA), an n-6 PUFA, are two common ligands for FABP7; however, FABP7’s affinity for DHA (K_D_ ∼ 10 nM) is approximately 4-fold higher than it is for AA (Xu et al., 1996). While FABP7 is also thought to bind the important n-3 PUFA eicosapentaenoic acid (EPA; Balendiran et al., 2000), little is known about the physiologic effect of FABP7-EPA binding, hence our focus on DHA as the primary n-3 ligand for FABP7. Upon binding DHA, FABP7 is reported to undergo a 3D conformational change that exposes a nuclear localization signal (NLS) and leads to FABP7-mediated transport of DHA to the nucleus (Ayers et al., 2007; Wolfrum, 2007). Once in the nucleus, DHA can trigger the expression of a range of anti-inflammatory genes via the activation of peroxisome proliferator-activated receptors (PPARs; Li et al., 2005; Kagawa et al., 2019). PPARs, divided into isotypes α, β/δ, and γ, are nuclear receptor proteins that function as transcription factors upon their binding of fatty acid ligands (Wolfrum, 2007; Crowder et al., 2017). FABPs are known to have varying affinities for the three PPAR isotypes, with FABP7 specifically interacting with and activating PPARγ (Mita et al., 2010; Tripathi et al., 2017). Genes under the control of PPARγ include anti-inflammatory (Martin, 2010) and neuroprotective targets (Kumar et al., 2020). Activation of PPARγ is also responsible for the anti-migratory phenotype seen in malignant glioma cell lines cultured with DHA (Mita et al., 2010; Elsherbiny et al., 2013).

In contrast, FABP7-mediated migration in malignant glioma is dependent upon the translocation of FABP7-bound AA to the ER to activate cyclooxygenase 2 (COX-2) dependent pro-migratory and pro-inflammatory pathways (Elsherbiny et al., 2013). COX-2 is an enzyme that plays a key role in the generation of inflammation via the conversion of AA to pro-inflammatory prostaglandins (particularly PGE_2_) as well as the production of pro-inflammatory chemokines and cytokines (Chen, 2010). Interestingly, PPARγ activation leads to the downregulation of COX-2 (Bren-Mattison et al., 2007), indicating potential autoregulatory feedback loops with reciprocal effects. By altering the expression level of FABP7 and the ratio of DHA:AA in malignant glioma cells, it was found that DHA and AA affect migration in an FABP7-dependent manner, with DHA inhibiting migration and AA promoting migration (Mita et al., 2010). Elsherbiny et al. (2013) later confirmed these results, suggesting that there is a deregulation of lipid homeostasis in malignant gliomas that significantly increases the ratio of AA:DHA and promotes migration.

Taken together, these findings lay out a dichotomous role for FABP7 in regulating both pro-inflammatory and anti-inflammatory pathways. More specifically, higher relative levels of DHA promote FABP7-mediated delivery of DHA to the nucleus, resulting in subsequent activation of PPARγ and the transcriptional activation of downstream neuroprotective and anti-inflammatory pathways, while, alternatively, higher relative levels of AA promote FABP7-mediated delivery of AA to the ER to interact with COX-2, leading to an increase in pro-inflammatory factors and a pro-migratory phenotype.

## Lipid Transport in Alzheimer’s Disease

Alzheimer’s disease is a progressive neurodegenerative disease resulting in neuronal death and cognitive decline. Pathologically, AD is characterized by the accumulation of intracellular neurofibrillary tangles (NFTs) and extracellular amyloid plaques (Breijyeh and Karaman, 2020). With a growing aging population and AD prevalence on the rise (Alzheimer’s Association, 2021), there has been a significant effort to characterize the molecular mechanisms underlying AD pathogenesis. Many researchers have investigated heritable forms of AD for clues as to what initiates AD neuropathies. Carriers of the ε*4* allele of the lipid binding protein apolipoprotein E (APOE) are currently at the highest risk of developing AD, with an estimated 40–80% of AD patients possessing at least one *APOE*ε*4* allele (Mahley et al., 2006). In the brain, APOE released by astrocytes and microglia binds essential lipids and delivers them to neurons via APOE receptors expressed on neuronal membranes (Holtzman et al., 2012). Since APOE is involved in lipid transport and metabolism, this has led many to hypothesize that lipid dysregulation plays a significant role in AD pathogenesis.

Alzheimer’s disease brains have been shown to have a high occurrence of intracellular lipid deposits, suggesting that aberrant lipid metabolism is a feature of AD (Foley, 2010; Di Paolo and Kim, 2011). However, such deposits may also result from abnormal lipid storage and/or transport. Lipid droplets (LDs) are lipid storage organelles with a neutral lipid core, mostly consisting of esterified cholesterol and triglycerides, surrounded by a monolayer of polarized lipids (Fujimoto and Parton, 2011; Welte, 2015; Cohen, 2018). LDs store lipids to be used for membrane structures, lipid signaling, and energy metabolism (Walther and Farese, 2012), and they are receiving increased attention for their role in neurodegenerative diseases, including AD (Farmer et al., 2020; Ralhan et al., 2021). Indeed, Alzheimer (1907) originally observed “adipose inclusions” in many glia in AD patients (Stelzmann et al., 1995), and tissues harvested from along the lateral ventricle in both human AD post-mortem brains and 3xTgAD mouse model brains have been shown to accumulate LDs (Hamilton et al., 2015).

Recent studies have shown that neuronal activity can initiate lipid peroxidation, lipoprotein export, and peroxidized lipid storage of LDs in astrocytes (Ioannou et al., 2019). Oxidative waste products from neurons have also been described to be transported to glia via apolipoproteins (Liu et al., 2017). The efficiency of this lipid shuttling is dependent upon the type of APOE isoform, with ApoE ε4 lipoproteins being less effective at transferring lipotoxic products to glia than ApoE ε3 lipoproteins. The ApoE ε4 isoform has also been associated with increased unsaturation of fatty acids and the accumulation of intracellular LDs, compared to the ApoE ε3 isoform, in both yeast and human iPSC-derived astrocytes (Sienski et al., 2021). Human astrocytes with excess triacylglycerol-laden LDs, a phenomenon associated with aging and stress, redirect ApoE toward LD secretion, which is exacerbated by ApoE ε4 (Lindner et al., 2022). ApoE ε4 also lacks cysteine residues that are present in ApoE ε2 and ApoE ε3 that are thought to scavenge the harmful lipid peroxidation end-product 4-hydroxynonenal (HNE), a highly reactive and neurotoxic molecule (Butterfield and Mattson, 2020). Various FABPs, such as epithelial and adipocyte FABPs, have also been shown to bind HNE (Bennaars-Eiden et al., 2002; Hellberg et al., 2010; Smathers and Petersen, 2011), but whether this occurs in FABPs expressed in the central nervous system remains to be explored.

A definitive relationship has been established between the lipid transport protein APOE and AD risk, but the role of FABP7 in AD remains less clear. Interestingly, FABP7 has been shown to protect astrocytes from reactive oxygen species toxicity through LD formation (Islam et al., 2019).

Moreover, recent evidence suggests that APOE and FABP7 may interact, with the APOE isoform determining the functional expression of FABP7 (Asaro et al., 2021). In mouse brains expressing the ApoE ε3 isoform (the most common isoform), this transporter delivers lipids to the neuronal receptor sortilin, which mediates the transfer of lipids from the exterior of the cell to the interior (Carlo et al., 2013). Sortilin directs the uptake and conversion of polyunsaturated fatty acids into endocannabinoids, lipid-based neurotransmitters that act through nuclear receptors to sustain neuroprotective gene expression in the brain (Asaro et al., 2020). In a mouse model, sortilin was also shown to promote the stability of FABP7 in an APOE isoform-dependent manner, with ApoE ε3 promoting the proper intracellular sorting of FABP7 and ApoE ε4 disrupting it (Asaro et al., 2021). APOE isoform-related differences in FABP7 have also been seen in humans (Asaro et al., 2021). Indeed, *APOE*ε*3/APOE*ε*3* patients were observed to have significantly higher levels of FABP7 than *APOE*ε*4/APOE*ε*4* patients, providing a novel connection between ApoE ε4 and FABP7 and suggesting that inhibition of FABP7 signaling may be one mechanism of ApoE ε4-induced AD development and/or progression.

In a proteomic screen of post-mortem AD brains, alterations in the levels of FABP5 and FABP7 were not observed, while FABP3 levels were significantly decreased (Cheon et al., 2003). However, another proteomic screening study of post-mortem AD brains found that both FABP3 and FABP7 levels were elevated in the brains of symptomatic AD patients compared to those of asymptomatic AD patients, with the increase of FABP7 being significantly higher than that of FABP3 (Johnson et al., 2018, 2020; Higginbotham et al., 2020; Hampel et al., 2021a; Rayaprolu et al., 2021; Wingo et al., 2021). Differences in the levels of FABP types outside the brain have also been observed in AD. Indeed, serum levels of FABP7, but not FABP3, were observed to be elevated in 29% of AD patients (Teunissen et al., 2011). Future studies in AD patients will be needed to establish clear patterns of brain and peripheral FABP7 expression levels, with consideration of which APOE isoform is expressed as well as the stage and symptomology of disease.

## Lipids and Inflammatory Pathways in Alzheimer’s

In addition to amyloid plaques and NFTs, altered inflammatory processes are thought to be another key hallmark of AD. Signs of inflammation were first noted by Alois Alzheimer in his initial description of the disease in 1907, though they were largely ignored due to the long-held thought that the brain is immunologically privileged (Alzheimer, 1907; Stelzmann et al., 1995; Akiyama et al., 2000). However, McGeer et al. (1987) reported that AD brains showed activated microglia expressing human leukocyte antigen – DR isotype (HLA-DR), an immunological marker previously associated exclusively with peripheral leukocytes. Given this link to inflammation, it was thought that those taking conventional non-steroidal anti-inflammatory drugs (NSAIDs) should have lower incidences of AD (McGeer et al., 1996). A series of epidemiological studies later confirmed AD sparing in patients consuming NSAIDs; however, this effect was only seen when the drugs were started at least 6 months prior to the clinical diagnosis of AD (McGeer et al., 1996). While the research efforts surrounding AD and inflammation tend to be highly compartmentalized, the neuroinflammation seen in AD is likely multifaceted, involving an array of inflammatory processes working in conjunction with one another (Akiyama et al., 2000).

The anti-inflammatory effects of many NSAIDs are dependent upon COX-2 inhibition or PPARγ activation (Jiang et al., 1998; Zarghi and Arfaei, 2011), suggesting that alterations in these pathways could be involved in the chronic neuroinflammation associated with AD. AA and DHA are known modulators of these inflammatory pathways (Higgins and Lees, 1984; Calder, 2010, p. 3) and thus may represent mechanisms linking FABP7 with glial inflammatory activation in neurodegenerative diseases. To test the responses to DHA and AA in AD, many have turned to animal models. In a mouse model of AD, a diet enhanced with AA was found to significantly increase Aβ burden, furthering disease progression (Amtul et al., 2012; Hosono et al., 2015). COX-2 is thought to convert AA into the inflammatory compound PGE_2_ in astrocytes and microglia, suggesting that increased AA levels may translate into increased inflammation via this pathway (Mohri et al., 2007). Conversely, DHA has been shown to display neuroprotective properties in AD. DHA first emerged as a compound of interest in AD research when fish consumption was linked to the decreased prevalence of AD (Grant, 1997; Horrocks and Yeo, 1999). Since then, more than 20 large-scale epidemiological cohorts have been used to study the relationship between PUFAs and AD, with many of them finding a negative correlation between DHA consumption and AD risk (Cederholm et al., 2013). These findings led to the development of many clinical trials studying the impact of DHA intervention on AD; however, these studies largely failed to establish DHA as a viable treatment option (Cunnane et al., 2013; Burckhardt et al., 2016).

While the epidemiological evidence supporting a beneficial role for DHA is robust, the lack of clinical trial success has made DHA a controversial topic among AD researchers (Cole et al., 2009; Pan et al., 2015; Radcliffe et al., 2016). Within animal models, however, early administration of DHA has shown positive effects in slowing the progression of AD (Lim et al., 2005; Cole and Frautschy, 2006, 2010; Perez et al., 2010; Arsenault et al., 2011; Park et al., 2020), suggesting that DHA may need to be given prior to clinical manifestations in order to be beneficial. Moreover, Arellanes et al. (2020) recently proposed that clinical trials of DHA may have failed to show positive effects on AD due to the dosages simply being too low. In a randomized, placebo-controlled clinical trial using 2,152 mg of DHA per day, there was a 28% increase in the cerebrospinal fluid (CSF) level of DHA in the treatment group compared to the placebo group (Arellanes et al., 2020). With previous studies having established that AD patients have decreased DHA levels in the brain (Conquer et al., 2000), this study suggests that supplementation may be able to delay disease progression. While the molecular mechanisms underlying DHA-mediated neuroprotection in AD are not well understood, we hypothesize that FABP7, which plays a key role in the trafficking of DHA, will emerge as an essential factor for mediating DHA’s neuroprotective effects.

## FABP7 Signaling and Neuroinflammation in Alzheimer’s Disease

**FIGURE 2:**
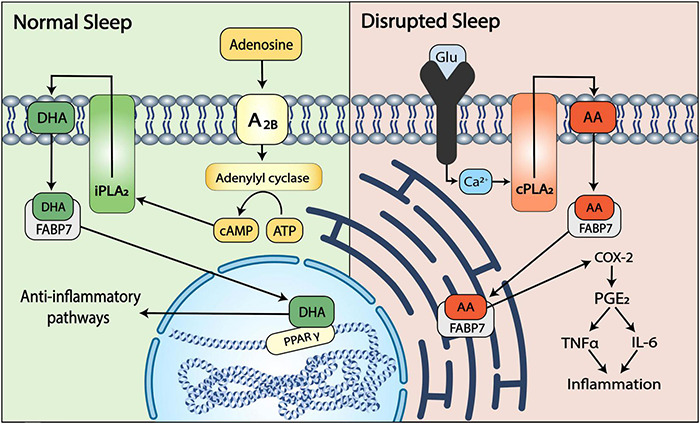
A dichotomous role for FABP7 in AD. Depending on the relative activity of cPLA_2_ and iPLA_2_, AA and DHA are released from the phospholipid membranes of astrocytes. Upon release, AA or DHA bind FABP7 to initiate distinct signaling cascades within the cell. Under conditions of normal sleep (left), the sleep promoting substance adenosine activates adenosine receptors (A2B) that signal adenylyl cyclase to increase cAMP levels, leading to the cAMP-dependent activation of iPLA_2_ and subsequent release of DHA from the membrane. The released DHA binds to FABP7 and is translocated to the nucleus where it activates the anti-inflammatory transcription factor PPARγ, leading to the induction of sleep-mediated neuroprotective pathways. With disrupted sleep (right), wakefulness-associated increases in glutamate result in increased levels of intracellular Ca^2+^ that activate cPLA_2_ to release AA from the membrane to bind FABP7. FABP7 then delivers AA to the endoplasmic reticulum, triggering a COX-2:PGE_2_-dependent pro-inflammatory cascade and cytokine (TNFα and IL-6) –mediated progression of neurodegeneration.

Based on the signaling cascades and mechanisms observed in cancer (described above), the increased level of FABP7 in AD brains, and the evidence for AA-mediated AD progression and DHA-mediated neuroprotection, we hypothesize that FABP7 serves a dichotomous role in neurodegeneration, with the relative AA:DHA ratio ultimately determining FABP7’s cellular function (Figure 2) and, consequently, AD pathogenesis and progression. The release of AA and DHA are dependent upon the phospholipases (PLAs; Elsherbiny et al., 2013) calcium-dependent PLA_2_ (cPLA_2_) and calcium-independent PLA_2_ (iPLA_2_), respectively (Chakraborti, 2003). Interestingly, elevated levels of cPLA_2_ immunoreactivity have been found in the astrocytes of post-mortem AD brains (Stephenson et al., 1996), and a genetic polymorphism in cPLA_2_ has been associated with late-onset AD (Cordeiro et al., 2010). These findings suggest that cPLA_2_ may play a role in the upstream initiation of neuroinflammation in our model. Given that cPLA_2_ acts on AA and iPLA_2_ acts on DHA in cell membranes, we propose that increased cPLA_2_ activity may be responsible for the increased AA availability and, consequently, increased inflammation observed in AD. More specifically, increased AA leads to FABP7-mediated translocation to the endoplasmic reticulum, where AA is converted to PGE_2_ by COX-2. PGE_2_ then triggers an inflammatory cascade by binding to one of its G protein-coupled E-prostanoid receptors (Ricciotti and FitzGerald, 2011). While an increase in the PGE_2_ receptor E-prostanoid 3 (EP3) has been linked to inflammation in AD (Shi et al., 2012), we provide a novel explanation for the mechanism underlying PGE_2_ production.

In addition to stimulating EP3, we suggest that AA-induced PGE_2_ production contributes to astrocytic inflammation in AD by elevating levels of the major inflammatory cytokine interleukin 6 (IL-6). IL-6 has been found at significantly increased levels in AD patients and has been suggested to be one of the major drivers of AD-linked neuroinflammation (Cojocaru et al., 2011; Wang W.-Y. et al., 2015; Wu et al., 2015; Lyra e Silva et al., 2021). We theorize that this increase in IL-6 may be mediated by PGE_2_ since PGE_2_ has been found to induce IL-6 synthesis in a human astrocytoma cell line (Fiebich et al., 1998), human synovial fibroblasts (Inoue et al., 2002), cultured astrocytes (Fiebich et al., 2001), and a murine model of inflammation (Hinson et al., 1996).

TNFα, another major inflammatory cytokine that is elevated in the blood of AD patients, may play an even larger role in not only the exacerbation of inflammation late in disease progression but also in the early stages of disease prior to diagnosis. In a study comparing the CSF levels of TNFα in patients with mild cognitive impairment (MCI) versus age-matched healthy controls, TNFα was markedly increased (*p* = 0.0009) in the patients with MCI (Tarkowski et al., 2003). Of the 56 patients with MCI that took part in the study, 31 had developed AD by the 9-month follow-up. When the initial CSF levels of TNFα in MCI patients that had and had not developed AD were separated and compared to control levels, only those that had developed AD showed significant increases compared to controls, suggesting that high TNFα levels may be an early marker for AD. This early rise in TNFα may indicate the beginning of an inflammatory cascade, as TNFα not only causes inflammation itself but has also been found to stimulate the COX-2 pathway, resulting in increased PGE_2_ levels (Zhang and Dziak, 1996). In our model, we suggest that FABP7-mediated translocation of AA to the ER leads to increased production of PGE_2_ and the subsequent generation of inflammation mediated by both IL-6 and TNFα.

Recent studies have highlighted the role of glial cells, specifically astrocytes and microglia, in disease-related inflammatory processes (Kaur et al., 2019; Nordengen et al., 2019; Hampel et al., 2020; Kwon and Koh, 2020; Leng and Edison, 2021). Microglia, the brain’s resident macrophages, have been hypothesized to play a role in regulating inflammatory changes as they perpetually survey the brain for pathogens, injuries, and other disturbances in the environment (Nayak et al., 2014; Colonna and Butovsky, 2017; Hansen et al., 2018). In the early stages of AD, microglia are able to keep amyloid beta (Aβ) plaques at bay by (a) phagocytosing Aβ (Koenigsknecht and Landreth, 2004; Simard et al., 2006; Bolmont et al., 2008; Pan et al., 2011), and (b) surrounding Aβ plaques, creating a physical barrier to prevent spreading and toxicity to neighboring regions (Condello et al., 2015). While these processes are initially sufficient to clear Aβ in the normal brain, there is thought to be a tipping point in AD pathogenesis at which microglia are no longer able to manage the Aβ burden and, for reasons that remain to be fully elucidated, become harmful to the brain (Aguzzi et al., 2013; Sarlus and Heneka, 2017; Hansen et al., 2018; Kaur et al., 2019).

Disease-associated microglia (DAM; Keren-Shaul et al., 2017; Deczkowska et al., 2018), an activated form of microglia, have been identified near Aβ plaques; however, the role they play in AD pathogenesis has been a topic of controversy, with some suggesting that they play a neuroprotective role (Jay et al., 2017; Keren-Shaul et al., 2017; Ulrich et al., 2017; Yeh et al., 2017) and others suggesting that they contribute to neurodegeneration (Colonna and Butovsky, 2017; Liddelow et al., 2017). More recent studies have indicated that the role of microglia in AD is less black and white, with a shift from neuroprotection to neurodegeneration occurring as the disease progresses. Genomic profiling studies of microglia in mice have revealed that the transition to DAM includes alterations in the expression of hundreds of genes (Orre et al., 2014; Wang Y. et al., 2015; Srinivasan et al., 2016; Keren-Shaul et al., 2017). As Aβ plaques accumulate, microglial genes related to homeostasis are downregulated, and genes known to play a role in neurodegeneration are upregulated (Hansen et al., 2018). While homeostasis genes are generally downregulated as AD progresses, there is an exception to this pattern. Among the genes whose expression are markedly increased in DAM is the homeostatic gene *TREM2* (Kamboh, 2018; Hampel et al., 2020), which encodes a microglial cell surface receptor that promotes microglial phagocytosis of Aβ (Guerreiro et al., 2013; Zhong et al., 2017; Zheng et al., 2018). Despite being upregulated in pro-inflammatory DAM, the most common mutation in *TREM2*, which is a loss of function mutation, confers a threefold increased risk of developing AD (Guerreiro et al., 2013; Jonsson et al., 2013, p. 2). TREM2 has also been shown to suppress the release of pro-inflammatory cytokines (Zhong et al., 2015; Zhang et al., 2017; Zhu et al., 2020), suggesting that it plays a neuroprotective role in the brain.

Despite its likely beneficial role, overexpression of TREM2 is insufficient to ward off AD progression. While there are likely a multitude of factors that contribute to microglial activation, lipopolysaccharide (LPS) associated with Aβ plaques has been hypothesized to trigger the mTOR pathway, causing microglia to release pro-inflammatory cytokines, including TNFα and IL-6 (Riazi et al., 2008; Welser-Alves and Milner, 2013; Wang Y. et al., 2015; Zhan et al., 2018). As shown above, these cytokines lead to neuroinflammation and are thought to contribute to neuronal dysfunction and death (Rothwell, 1999; Aktas et al., 2007; Wang Y. et al., 2015; Kempuraj et al., 2017). They can also upregulate β-secretase, an enzyme that cleaves amyloid precursor protein (APP) to create aggregation-prone Aβ (Cole and Vassar, 2007; Hampel et al., 2021b), further perpetuating AD progression. Additionally, cytokines released by microglia, particularly IL-1α, TNFα, and C1q, have been shown to cause astrocytes to transform into a reactive state called A1 (Liddelow et al., 2017). A1 reactive astrocytes then release pro-inflammatory cytokines, creating further neuronal damage and contributing to AD pathogenesis. The mechanism for microglia-induced astrocytic activation is not fully understood; however, we propose that FABP7 may play a role since TNFα has been shown to induce cPLA_2_ expression (Yang et al., 2014), which we hypothesize sets off an FABP7-dependent pro-inflammatory cascade.

## Alzheimer’s Disease, FABP7, And Sleep

Although AD is best known for its detrimental impact on memory, sleep disturbances are another common feature of the disease, with epidemiological data suggesting that such interruptions are experienced by up to 45% of AD patients (McCurry et al., 2000; Moran et al., 2005; Peter-Derex et al., 2015). Sleep deficits are known to worsen with disease progression, often resulting in early institutionalization; however, more recent evidence suggests that sleep disturbances may arise years before cognitive deficits (Ju et al., 2013; Lim et al., 2013; Zhang et al., 2019; He et al., 2020; Lloret et al., 2020). While the relationship between AD and sleep disturbances is complex and bi-directional (Lucey, 2020), the early appearance of changes in sleep suggests that sleep disturbances may be a useful prodromal marker for AD.

In addition to its role in lipid transport and metabolism, FABP7 plays an important role in the regulation of normal sleep in multiple species including flies, mice, and humans (Gerstner et al., 2017b). FABP7 has also been shown to have diurnal regulation in the astrocytes of adult rodents (Gerstner et al., 2008). Of particular relevance to AD, FABP7 induction was found to enhance both sleep and long-term memory consolidation in flies (Gerstner et al., 2011a,b), two processes that are consistently dysregulated in AD (Musiek et al., 2015). Furthermore, there seems to be a positive feedback loop between fragmented sleep and AD progression in which fragmented sleep leads to an accumulation of Aβ, and Aβ accumulation, in turn, leads to more fragmented sleep (Gerstner et al., 2012a; Roh et al., 2012; Ju et al., 2014; Lim et al., 2014; Musiek and Holtzman, 2016). Previously, we proposed an astrocyte-specific mechanism for this feedback loop (Vanderheyden et al., 2018) in which age-associated sleep decline decreases Aβ clearance, leading to the formation of Aβ plaques that act as “sinks” for Aβ oligomers. It is thought that these “sinks,” along with the concentration gradient of oligomers around them, attract glia-mediated clearing mechanisms, resulting in the dysregulation of the astrocyte-neuron-lactate-shuttle (ANLS), a system that normally serves to regulate the metabolic demands of neurons via lactate release (Pellerin and Magistretti, 1994, 2012; Petit et al., 2013), which is closely tied to glutamate release. With the uncoupling of the ANLS, excess glutamate accumulates and causes increased Aβ release, excitotoxicity, and wakefulness that perpetuates the cycle (Vanderheyden et al., 2018).

Alongside uncoupling of the ANLS, we propose that FABP7-mediated subcellular localization in astrocytes is directly influenced by sleep, with FABP7-dependent nuclear translocation of DHA promoting healthy, sleep-mediated outcomes, and wakefulness driving the translocation of FABP7-AA to the ER, thus promoting pathological outcomes. This FABP7-DHA-mediated activation of anti-inflammatory pathways includes the inhibition of NF-κB (Lee et al., 2009; Zgórzyńska et al., 2021), a transcription factor responsible for the upregulation of COX-2 expression. COX-2 converts AA into PGE_2_ (Kim et al., 2009), which is known to play a role in regulating the sleep-wake cycle (Hayaishi, 1988; Matsumura et al., 1989), and within astrocytes specifically, it promotes wakefulness via the stimulation of glutamate release (Bezzi et al., 1998). Therefore, we suggest that FABP7-DHA nuclear translocation results in decreased NF-κB expression, which, in turn, leads to decreased expression of COX-2 and decreased production of wake-promoting PGE_2_, ultimately resulting in an increase in sleep. Interestingly, improved sleep quality has been correlated with reduced NF-κB levels in older adults (Black et al., 2015), implying that sleep is beneficial not only for the clearance of accumulated Aβ but also for promoting the downregulation of wake-promoting PGE_2_. Additionally, a placebo-controlled, double-blind study found that healthy adults consuming an oil rich in DHA had significantly improved sleep efficiency and latency compared to those taking a placebo (Patan et al., 2021), further suggesting that DHA levels play an important role in sleep quality.

Conversely, we propose that FABP7-mediated delivery of AA to the ER promotes wakefulness, via the conversion of AA to PGE_2_ by COX-2 (Mohri et al., 2007). PGE_2_ was found to upregulate NF-κB in a macrophage cell line (Camandola et al., 1996), suggesting a feedback loop in which high levels of AA promote COX-2-mediated production of PGE_2_. PGE_2_ then upregulates NF-κB, and NF-κB, in turn, upregulates COX-2, leading to even more PGE_2_. Along with upregulating COX-2, NF-κB is responsible for promoting the transcription of pro-inflammatory cytokines and chemokines that have been proposed to induce cellular damage as well as stimulate Aβ release in astrocytes (Shi et al., 2016; González-Reyes et al., 2017). As previously mentioned, an increase in Aβ release is thought to promote wakefulness (Musiek et al., 2015; Vanderheyden et al., 2018), raising the possibility that AA-mediated activation of NF-κB further perpetuates sleep disturbances in AD. While the specific effect of AA on sleep has not been studied, sleep disruptions have been shown to lead to the overexpression of NF-κB in humans (Angelo et al., 2014), supporting the hypothesis that early alterations in sleep trigger a positive feedback loop in which more wakefulness, Aβ accumulation, and inflammation occur.

Docosahexaenoic acid and AA are released from the cell membrane by iPLA_2_ and cPLA_2_, respectively (Elsherbiny et al., 2013). One known activator of cPLA_2_ is the excitatory neurotransmitter glutamate (Kim et al., 1995; Hartz et al., 2019). Given that glutamate is associated with wakefulness (Watson et al., 2011), we hypothesize that sleep disruption triggers a cascade that ultimately leads to excessive PGE_2_ production. PGE_2_ increases both COX-2 expression (Hsu et al., 2017) and wakefulness (Matsumura et al., 1989), setting up a vicious cycle in which sleep deprivation leads to the production of pro-inflammatory PGE_2_, and PGE_2_ leads to more wake. A study in healthy adults found that 88 h of total sleep deprivation induced a 30% increase in PGE_2_ (Haack et al., 2009), demonstrating that increased wakefulness does in fact increase PGE_2_ *in vivo*. We propose a mechanism for this increase in which wakefulness increases glutamate levels, which activates cPLA_2_ to release AA from the membrane, thus allowing FABP7 to deliver AA to the ER where it is converted into PGE_2_ (Figure 2).

In addition to the relationship between sleep disturbance and AA release, we propose that normal sleep promotes the activation of iPLA_2_, leading to increased DHA release, FABP-mediated translocation of DHA to the nucleus, and the subsequent activation of anti-inflammatory pathways. Cyclic adenosine monophosphate (cAMP), a compound thought to increase during sleep and decrease during sleep deprivation (Vecsey et al., 2009), has been shown to activate iPLA_2_ (Strokin et al., 2003), providing a basis for the finding that sleep is neuroprotective (Eugene and Masiak, 2015; Schneider, 2020). The levels of cAMP present during sleep may also have larger implications for the memory issues seen in AD. Indeed, Havekes et al. (2014) showed that sleep deprivation-associated memory deficits could be prevented by transiently increasing cAMP levels during periods of sleep loss, and others have proposed cAMP enhancers as potential therapeutic agents for AD (De Felice et al., 2007).

## Alzheimer’s Disease, FABP7, And the Circadian Clock

Alongside sleep disturbance, another mechanism that may influence FABP7-signaling in AD is disruption of the circadian system. Circadian expression of *Fabp7* is regulated by the core-clock gene BMAL1 (Gerstner and Paschos, 2020) and the circadian transcriptional repressor REV-ERBα (NR1D1) (Vanderheyden et al., 2021) and exhibits time-of-day changes at the tripartite synapse (Gerstner et al., 2012b). Many studies in humans and in animal models have shown a link between the circadian clock and AD (Weldemichael and Grossberg, 2010; Cermakian et al., 2011; Musiek and Holtzman, 2016; Homolak et al., 2018; Wu et al., 2019; Lananna and Musiek, 2020; Carter et al., 2021; Fusilier et al., 2021; Nassan and Videnovic, 2022). For example, gene ablation of *Bmal1* in mice has been shown to exacerbate amyloid burden and astrogliosis (Musiek et al., 2013; Kress et al., 2018). It is interesting to note that a recent study showed that plaque burden was unaffected in astrocyte-specific *Bmal1* knockout mice, but these mice still exhibited increased *Fabp7* gene expression and astrogliosis (McKee et al., 2022). In contrast, pharmacological activation of REV-ERB using the agonist SR9009 was shown to reverse cognitive decline and Aβ burden in the SAMP8 mouse model of AD (Roby et al., 2019). However, in another study, inhibition of REV-ERBs using the drug SR8278 or via gene knockdown was shown to promote microglial clearance of Aβ in 5XFAD mice (Lee et al., 2020). Such discrepancies between studies may be due to differences in cell-type, the particular AD animal model, or the specificity/efficacy of the of the drug/knockdown strategies used. Given that our model supports a dichotomous role for FABP7, either increasing or decreasing its expression may have different consequences depending on the relative levels of DHA:AA present in the cell (Figure 2). Future studies focused on determining the molecular-genetic interactions of the circadian system with AD-related pathophysiology in glial cells will be important for our understanding of AD progression and etiology.

## Astrocytes and Alzheimer’s Disease Etiology

Astrocytes were once thought to merely support the metabolic needs of neurons due to their lack of action potentials. However, there is now clear evidence demonstrating that astrocytes play active and critical roles in the functioning of the central nervous system (Somjen, 1988; Agulhon et al., 2008), and these cells are receiving more attention in the treatment of neurodegenerative diseases (Finsterwald et al., 2015; Valori et al., 2021). Astrocytes respond to external stimuli by modulating intracellular calcium levels and releasing neurotransmitters in a phenomenon known as gliotransmission (Volterra and Meldolesi, 2005). Additionally, neurons and astrocytes are now understood to be integrated through the tripartite synapse, a model demonstrating that astrocytes surround neuronal junctions, taking part in both pre- and post-synaptic activities (Araque et al., 1999; Halassa et al., 2009; Perea et al., 2009; Papouin et al., 2017). More recent evidence indicates crucial metabolic cooperation between astrocytes and neurons in which glycolytic metabolism in astrocytes produces metabolites such as L-lactate and L-serine, which are then shuttled to neurons to meet their high metabolic needs (Bonvento and Bolaños, 2021). Among many other factors, the disruption of this metabolic relationship may be a very early contributor to the synaptic dysfunction seen in AD (Demetrius and Driver, 2013; Gordon et al., 2018; Bonvento and Bolaños, 2021).

Reactive astrocytes, astrocytes that have undergone molecular and functional changes in response to pathological conditions, have been detected in the early stages of AD before neuronal death (Perez-Nievas and Serrano-Pozo, 2018; Li et al., 2019). Given that there are high levels of these cells localized around amyloid plaques and that they persist throughout AD progression, reactive astrocytes have been suggested as a hallmark sign of AD (Pike et al., 1995; Chun and Lee, 2018). One of the most pertinent changes that occurs in reactive astrocytes is an increase in spontaneous calcium signaling, which ultimately impacts gliotransmission (Takano et al., 2007; Kuchibhotla et al., 2009; Delekate et al., 2014; Nanclares et al., 2021). This increase in calcium levels may be due to the upregulation of astrocytic neurotransmitter receptors, including glutamate receptors (Teaktong et al., 2003; Yu et al., 2005). This is particularly relevant to sleep and AD dynamics, as a system of communication between neurons and astrocytes has been proposed in which glutamate, which is associated with wakefulness, binds to astrocytic neurotransmitter receptors, triggering gliotransmission (Nanclares et al., 2021). In this model, glutamate activates G_q_ G-protein-coupled receptors (GPCRs) on the surface of astrocytes, triggering phospholipase C (PLC) to hydrolyze the membrane lipid phosphatidylinositol 4,5-bisphosphate (PIP_2_) into diacylglycerol (DAG) and inositol triphosphate (IP_3_; Agulhon et al., 2008). IP_3_ then acts as a second messenger, stimulating IP_3_ receptors and leading to the release of calcium from the ER. This internal rise in calcium, in turn, causes astrocytes to release gliotransmitters, including glutamate, which can then bind to nearby neurons (Scemes, 2000; Parri and Crunelli, 2003; Volterra and Steinhäuser, 2004; Fiacco and McCarthy, 2006; Scemes and Giaume, 2006; Malarkey and Parpura, 2008; De Pittà et al., 2009).

Normally, this neuron-astrocyte communication serves to maintain glutamate homeostasis by controlling glutamate uptake and release (Mahmoud et al., 2019); however, this balance is thought to be disrupted in AD, leading to an accumulation of glutamate (Nanclares et al., 2021), which may contribute to AD formation in several ways. First, increased glutamate has primarily been associated with wakefulness (Dash et al., 2009; Naylor et al., 2011; Lucey and Bateman, 2014), which may be exacerbated in pre-clinical AD (Vanderheyden et al., 2018); thus, this alteration in astrocytic gliotransmission may be one of the drivers of AD-associated insomnia. Second, under normal conditions, Aβ peptides are thought to be deposited during periods of wakefulness and cleared during sleep (Gerstner et al., 2012a; Roh et al., 2012; Hablitz et al., 2020). However, with decreased sleep, amyloid peptides may not be effectively cleared and may instead aggregate, forming the protein plaques that are the primary hallmark of AD. Recently, the clock modulator nobiletin was shown to mitigate astrogliosis and inflammation in an AD model (Wirianto et al., 2022). Third, in addition to sleep/circadian factors, increased glutamate release from astrocytes could lead to excitotoxicity, or cell damage/death, from excessively high levels of excitatory neurotransmission (Lewerenz and Maher, 2015). Finally, there is evidence demonstrating that there is an increase in cPLA_2_ immunoreactivity in reactive glia associated with Aβ deposits (Stephenson et al., 1996, 1999; Colangelo et al., 2002; Moses et al., 2006; Schaeffer and Gattaz, 2008), and that astrocytic calcium release activates cPLA_2_, which then stimulates AA release from the membrane (Stephenson et al., 1994; Sergeeva et al., 2005; Wang et al., 2021). This release would promote more binding of AA by FABP7 and subsequent initiation of the pro-inflammatory cascade described in our model.

## Treatment Strategies Targeting Diet and Lipid-Signaling Cascades for Alzheimer’s Disease

There is significant evidence that the downstream inflammation-determining PUFAs can be modulated through diet (Sharma et al., 2012; Thomas et al., 2015). Diet was first suggested to play a role in AD when a 1997 study found that elderly African-Americans and Japanese people living in the United States had significantly increased rates of AD compared to people from their countries of origin (Grant, 1997). Since this initial finding, diet has been repeatedly linked to AD prevalence, with evidence showing that increased omega-3 fatty acid consumption is significantly associated with a lower risk of AD (Morris, 2009). Accordingly, a Mediterranean diet has been associated with a lower risk of AD (Scarmeas et al., 2006), while a Western diet has been associated with an increased risk of AD (Berrino, 2002). The dietary balance between n-3 and n-6 PUFAs may explain the societal differences in AD prevalence, as the Mediterranean diet is rich in DHA from olive oil, fish, and vegetables, and the Western diet is high in AA from corn and vegetable oils (Zivkovic et al., 2011). Indeed, the anti-inflammatory and neuroprotective properties of DHA have shown promising epidemiological results, suggesting that DHA, or perhaps DHA analogs, could serve as useful therapeutics for AD moving forward.

Dietary conditions with high fat/high sugar content, such as the Western diet, are associated with the development of hyperglycemia and diabetes and may be closely related to dementia and AD (Lee et al., 2018). Along these lines, thiazolidinedione drugs (TZDs; also called glitazones), a class of pharmacological agents originally developed to treat insulin resistance and diabetes (Lebovitz, 2019), have shown promise for treating AD (Pérez and Quintanilla, 2015; Saunders et al., 2021). Glitazones are high affinity ligands for PPARγ and are known to reduce Aβ in various models (Camacho et al., 2004; Heneka et al., 2005; Liu et al., 2013; Gad et al., 2016; Quan et al., 2019) as well as tau pathology (Escribano et al., 2010; O’Reilly and Lynch, 2012; Cho et al., 2013; Hamano et al., 2016; Moosecker et al., 2019). Moreover, we have recently shown that glitazone treatment rescues phenotypic deficits and autophagy pathways in flies with mutations in the human ortholog of the glucocerebrosidase gene (GBA) (Shola-Dare et al., 2021), the strongest genetic risk factor for Parkinson’s disease (Schapira, 2015; Do et al., 2019; Avenali et al., 2020). Indeed, glitazone treatment in GBA mutant flies restored normal levels of Ref(2)P, the fly p62 homolog and marker of autophagic flux, suggesting that the PPARγ-pathway may represent a common therapeutic target for multiple neurodegenerative diseases (Kumar et al., 2020; Jamwal et al., 2021). Whether glitazones bind FABP7 to initiate FABP7-dependent neuroprotective signaling cascades is a future direction of study, and may represent a drug-target mechanism of glitazone activation of PPARγ for the treatment of AD.

In addition to PPARγ, PPARα has also been implicated in AD pathogenesis. PPARα is part of the greater PPAR receptor family and has been found to play roles in lipid metabolism and anti-inflammatory pathways (Vallée and Lecarpentier, 2016). In AD, PPARα is involved in the regulation of beta-secretase (BACE-1), an enzyme responsible for the cleavage and later formation of Aβ plaques, and studies have shown a significant downregulation of PPARα in AD pathology (Wójtowicz et al., 2020). Furthermore, treatment with the PPARα agonist gemfibrozil together with retinoic acid (GFB-RA) was recently shown to lower amyloid plaque burden, improve memory, and reduce astrogliosis in mice (Chandra and Pahan, 2019; Raha et al., 2021). These effects appear to be mediated by astrocytes through lysosomal biogenesis and autophagy pathways (Raha et al., 2021), although alternative mechanisms of PPAR-regulated activity, such as molecular transrepression, may also be involved (Ricote and Glass, 2007; Pawlak et al., 2014). GFB-RA has also been shown to stimulate two succeeding neuroprotective pathways that result in the degradation of beta-amyloid plaques. These pathways include (1) activation of the transcription factor EB (TFEB) and (2) activation of the low-density lipoprotein receptor (LDLR; Raha et al., 2021). LDLR knockout mice treated with GFB-RA were shown to have significantly decreased levels of astrocytic Aβ plaque uptake, suggesting an important role for LDLR in the transport of Aβ (Raha et al., 2021). In the 5XFAD mouse model of AD, GFB-RA treatment was also shown to improve autophagic flux by restoring normal levels of p62 (Raha et al., 2021), a ubiquitin-binding scaffold protein that is degraded by autophagy (Bjørkøy et al., 2009). Combined, these data implicate PPARs as critical players in the regulation of Aβ degradation and highlight the need for further research into the underlying mechanisms.

We have also shown that fly FABP or murine FABP7 overexpression rescues sleep deficits in a fly model of AD (Gerstner et al., 2017a). Given that FABPs are targets of PPAR agonists (Guan et al., 2001; Tan et al., 2002), future studies characterizing the role of FABPs in PPAR-mediated signaling pathways will be important for determining their potential as drug targets for the development of treatments for neurodegenerative diseases such as AD.

## Conclusion

Alzheimer’s disease has become a growing global health crisis in recent years, with the number of dementia cases expected to rise to 152 million by 2050 (AAIC, 2021). While clinical trials have been evaluating AD drugs since the early 1980’s, they have largely failed to establish efficacy (Schneider et al., 2014; Cummings et al., 2018; Yiannopoulou et al., 2019; Oxford et al., 2020). Evidence suggests that the pathological hallmarks of AD begin appearing up to 20 years before symptoms develop (Reiman et al., 2012; Dubois et al., 2014, 2016; Guennewig et al., 2021), raising the idea that clinical trials may have failed due to the administration of therapeutics too late in disease progression. Given that the damage done to the brain in the late stages of AD is largely irreversible, the identification of prodromal markers as well as novel therapeutic agents is critically important.

In the search for modifiable risk factors related to AD, both sleep disturbances and inflammation have gained recognition (Irwin and Vitiello, 2019). While inflammation and sleep have long been thought of as distinct processes within AD pathogenesis, more recent studies have found an association between poor sleep quality and increased systemic inflammation (Irwin et al., 2016; Irwin and Opp, 2017), implying interplay between these two factors. Here, we suggest that FABP7 may be a key player in the linkage between neuroinflammation and sleep disturbances in AD. Evidence suggests a bidirectional relationship between sleep and AD in which AD decreases sleep quality, and decreased sleep quality increases AD progression (Gerstner et al., 2012a; Roh et al., 2012; Ju et al., 2014). This decrease in sleep has been shown to increase Aβ release (Shokri-Kojori et al., 2018) as well as the release of glutamate (Talantova et al., 2013), an excitatory neurotransmitter that activates the lipid-releasing enzyme cPLA_2_ (Kim et al., 1995; Hartz et al., 2019). In our model, this cPLA_2_ activation leads to increased AA release from astrocytic membranes, allowing FABP7 to transport AA to the ER where it is converted to PGE_2_ by COX-2. PGE_2_ then promotes neuroinflammation (Hein and O’Banion, 2009) by increasing the expression of NF-κB and COX-2 (Hsu et al., 2017) and promotes wakefulness by stimulating further glutamate release (Bezzi et al., 1998), both of which contribute to the pathogenesis of AD (Wang et al., 2014; Woodling et al., 2016; Madeira et al., 2018; Sadeghmousavi et al., 2020). Alternatively, sleep seems to be neuroprotective; reducing neuroinflammation by downregulating the expression of NF-κB (Black et al., 2015). We suggest that this pathway can be stimulated by DHA in an FABP7-dependent manner. Following adenosine signaling-induced activation of iPLA_2_ and the subsequent release of DHA from the astrocytic membrane (Strokin et al., 2003), we propose that FABP7 binds the released DHA and transports it to the nucleus, where it both activates the anti-inflammatory transcription factor PPARγ and decreases the expression of NF-κB; thus preventing the development and progression of AD. While our hypothesis is centered around AD, we hope that the pathways described here will help to elucidate those underlying other FABP7-linked conditions including cancer, Down’s syndrome, schizophrenia, amyotrophic lateral sclerosis (ALS), and Parkinson’s disease.

## Summary

Building on the signaling cascades and mechanisms seen in cancer, we propose a novel role for FABP7 in sleep and AD pathogenesis in which ligand availability determines FABP7’s function, with DHA promoting neuroprotection and normal sleep, and AA promoting disrupted sleep and AD pathogenesis. Here, we suggest that the release of both DHA and AA are regulated by upstream sleep-dependent mechanisms. We propose that disrupted sleep causes the accumulation of the wake-promoting neurotransmitter glutamate, which then leads to the activation of cPLA_2_ and the release of AA from astrocytic membranes, making AA available to FABP7 for transport to the ER. Alternatively, normal sleep, promoted by the somnogen adenosine signals an increase in cAMP levels via adenylyl cyclase, activating iPLA_2_ to release DHA from astrocytic membranes and making DHA available to bind FABP7 for transport to the nucleus. We hypothesize that sleep disturbances perpetuate a cycle in which AA is released and transported to the ER, leading to the upregulation of COX-2 and the subsequent production of pro-inflammatory cytokines, including IL-6 and TNFα. This would contribute to the development and progression of AD since astrocytic inflammation leads to uncoupling of the ANLS, which is known to cause subsequent increases in Aβ release. While further studies are required to confirm the mechanisms proposed in our model, investigations into the relationship between sleep and AD will provide a crucial new lens through which to understand the development of AD and could lead to the development of novel therapeutic strategies.

## Data Availability Statement

The original contributions presented in this study are included in the article/supplementary material, further inquiries can be directed to the corresponding author.

## Author Contributions

JG conceived the hypothesis. HN and JG wrote the first draft of the manuscript. All authors contributed to the manuscript revisions, read, and approved the submitted version.

## Conflict of Interest

The authors declare that the research was conducted in the absence of any commercial or financial relationships that could be construed as a potential conflict of interest.

## Publisher’s Note

All claims expressed in this article are solely those of the authors and do not necessarily represent those of their affiliated organizations, or those of the publisher, the editors and the reviewers. Any product that may be evaluated in this article, or claim that may be made by its manufacturer, is not guaranteed or endorsed by the publisher.
